# Classification of Wood Chips Using Electrical Impedance Spectroscopy and Machine Learning

**DOI:** 10.3390/s20041076

**Published:** 2020-02-17

**Authors:** Markku Tiitta, Valtteri Tiitta, Jorma Heikkinen, Reijo Lappalainen, Laura Tomppo

**Affiliations:** Department of Applied Physics, University of Eastern Finland, 70210 Kuopio, Finland; valtti@student.uef.fi (V.T.); jorma.heikkinen@uef.fi (J.H.); reijo.lappalainen@uef.fi (R.L.); laura.tomppo@uef.fi (L.T.)

**Keywords:** wood chips, machine learning, electrical impedance spectroscopy, scots pine, birch

## Abstract

Wood chips are extensively utilised as raw material for the pulp and bio-fuel industry, and advanced material analyses may improve the processes in utilizing these products. Electrical impedance spectroscopy (EIS) combined with machine learning was used in order to analyse heartwood content of pine chips and bark content of birch chips. A novel electrode system integrated in a sampling container was developed for the testing using frequency range 42 Hz–5 MHz. Three electrode pairs were used to measure the samples in x-, y- and z-direction. Three machine learning methods were used: K-nearest neighbor (KNN), decision tree (DT) and support vector machines (SVM). The heartwood content of pine chips and bark content of birch chips were classified with an accuracy of 91% using EIS from pure materials combined with a k-nearest neighbour classifier. When using mixed materials and multiple classes, 73% correct classification for pine heartwood content (four groups) and 64% for birch bark content (five groups) were achieved.

## 1. Introduction

The variety of solid bio-based raw materials has rapidly extended during the last decade. This means also a large variation of quality and specific properties. Wood chips are extensively utilised as a raw material for many bio-refining industrial processes, including bio-energy production, pulp and liquid bio-fuel industry. High quality wood chips used for pulp production are commonly known as pulp chips. If the properties of chip materials are known beforehand, the processes may be improved, for example by adjusting the amount of chemicals. Large amount of resins or bark may cause problems in bio-refining processes.

To the extent of our knowledge, there is no feasible on line technique to determine the extractive content of wood chips [1,2,3,4,5]. Perhaps the main challenge in the measurement is the inhomogeneous nature of the material and temperature variation starting below zero in Northern countries. Near-infrared/infrared (NIR/IR) techniques have been used for laboratory analyses of extractives and for on line moisture content (MC) measurement of wood chips. The drawback of the techniques is that often the calibration is very specific and only the surface layer can be measured. On the contrary, it is possible to obtain information from a relevant volume of biomass by EIS technique, and not only from the surface.

Electrical impedance spectroscopy (EIS) has been widely used to study different types of biological materials [6]. One of the main applications has been to study fundamental electrical properties of materials and correlate these properties with material structure. It may be used to investigate the dynamics of bound or mobile charge in the bulk or interfacial regions of liquid or solid materials (e.g., ionic or insulator materials). Many processes take place throughout the material when it is electrically stimulated and lead to the overall electrical response. A general EIS-based material-electrode system may be analysed using an exact mathematical model based on a plausible physical theory that predicts theoretical impedance or by an equivalent circuit model. In impedance spectroscopy analysis, the changes in concentrations between different types of charge carriers and the effect of changing microstructure are compared with impedance responses, and different types of electrical models are studied for describing the effect. An emphasis is put on the models with distributed elements, which can be used for estimation of different types of resistance and capacitance distributions.

With the EIS method, electrodes are used to create dynamic electric field in material, measure the complex electrical impedance at a specified frequency range and determine the properties of material in the effective electric field. The results may be utilised in further processing. Thus, it is possible to classify the sample according to its properties at an early stage of processing in forest industries, in order to increase the efficiency of wood processing and use.

The effect of various properties of wood on EIS has been widely studied [7,8,9,10,11,12,13]. EIS has been applied to tree physiology research, for example, the cell structure and temperature acclimation of trees have been extensively studied [14,15,16], but also other studies concentrating on physiology based factors have been published [17]. The distributed element circuit-based models were found to be most useful in the analyses [18]. Low frequency EIS has been used for wood decay, pore distribution and oil content analyses [19,20]. Recent studies include EIS applications for roots and wood-based composites [21,22].

The extractive content is significantly higher in birch bark compared with birch stem wood [23]. Pine heartwood contains substantially higher amounts of extractives compared to pine sapwood. Extractives of pine heartwood have a significant effect on electrical properties and they can be analysed using EIS [9,10,24,25,26]. The correlation is clear both in fresh and dried wood and according to the earlier studies, resin acid content may be determined using EIS.

Many classification and machine learning methods have been used for measurement data analyses. Recent studies include machine learning with optics, NIR and X-rays [27,28,29,30,31,32]. Electromagnetic waves using radio and microwave frequencies have been used with machine learning based classification techniques [33,34]. The results show the potential of machine learning combined with electromagnetic wave-based techniques for efficient non-destructive classification of biological materials.

Machine learning methods have also been applied for wood material classification. Successful classification analyses using optics, NIR, microwaves and ultrasonic data have been published [35,36,37,38,39]. In a recent study, moisture content of wood chips has been studied using electro-magnetic waves and support vector machines [40]. The most often used machine learning methods include Bayes, neural networks, supported vector machine, decision tree and k-nearest neighbor classifiers. Typically supervised classification methods have been used, including training and testing sets. Furthermore, deep learning has been used for wood material classification [40]. 

To the best of our knowledge, the heartwood content or bark content of wood chips has not been studied with EIS. In this study, EIS measurements were carried out in laboratory to analyse and classify bio-based raw material properties including bark/stem wood and sapwood/heartwood ratios. The main hypothesis was that with EIS and machine learning, these ratios could be determined, regardless of the MC and density variations, which are known to affect the electrical properties of wood. The underlying objective is to measure these ratios in the industry. This study is a preliminary step towards developing an industrial on line measurement.

## 2. Materials and Methods

**Figure 1:**
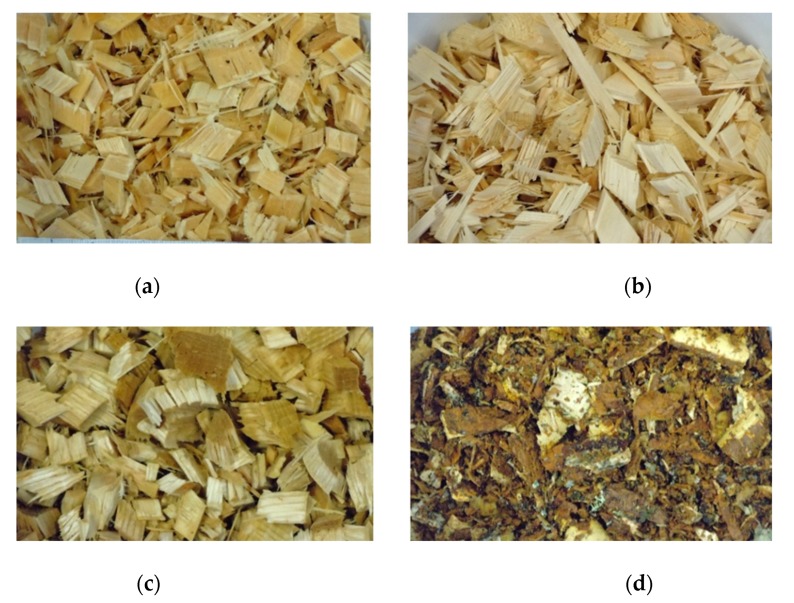
Studied materials: (**a**) Pine sapwood; (**b**) Pine heartwood; (**c**) Birch chips; (**d**) Birch bark.

Birch (Betula pendula/Betula pubescens) and Scots pine (Pinus Sylvestris) logs were collected from Northern Savo region in Finland in winter 2018. The pine material was cut from five logs including both sapwood and heartwood. The sapwood and heartwood parts of the logs were separated and the parts were cut to chips using Bosch AXT 25 TC shredder (Robert Bosch GmbH, Gerlingen-Schillerhöhe, Germany). The heartwood and sapwood materials were blended carefully to obtain samples with heartwood content of 100%, 75%, 50%, 25% and 0%. From the six birch logs, the bark was separated from the stem wood. Then the materials were mixed to get birch chip specimens with bark content of 100%, 75%, 50%, 25%, 0%. The pure materials are shown in Figure 1. The size distribution of the wood samples were similar, but the bark samples included more small particles (Figure 1).

Electrical impedance (*Z*) can be considered a complex quantity, which consists of real (resistance *R*) and imaginary parts (reactance *X*). Reactance can be represented in two forms, inductive and capacitive. The impedance plane representation can be plotted as separated functions of frequency (e.g., capacitance and conductance spectra) or in a complex plane with frequency as the parametric variable. The measurements can be presented in the impedance plane by plotting the imaginary part as a function of the real part.

The impedance measurements were carried out in a container (polypropylene, wall thickness 1.6 mm) with the measurement electrodes attached on the walls of the container. Prior to the impedance spectrum measurements, the measurement container was weighed with a plastic bag (polyethylene, thickness 10 µm) without sample chips. Then, the plastic bag in the container was filled with chips and weighed again. The plastic bag was first put into the container and then filled carefully with the tested material until the container was full. After filling, the lid was installed. Temperature (T) and relative humidity (RH) were measured, too. The measurements were conducted with normal laboratory conditions (T 22–24 °C; RH 25–40%). Weighing was used in determination of moisture content but not in further analyses.

**Figure 2:**
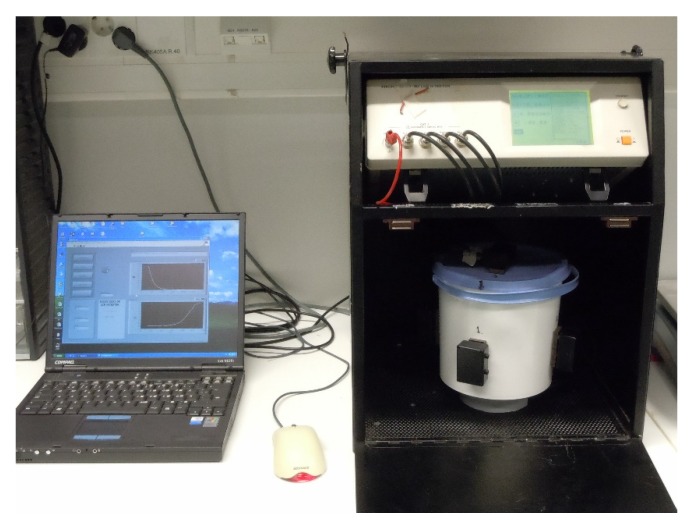
EIS measurement system: Portable PC, Hioki 3531Z HiTester impedance analyser and measurement container.

**Figure 3:**
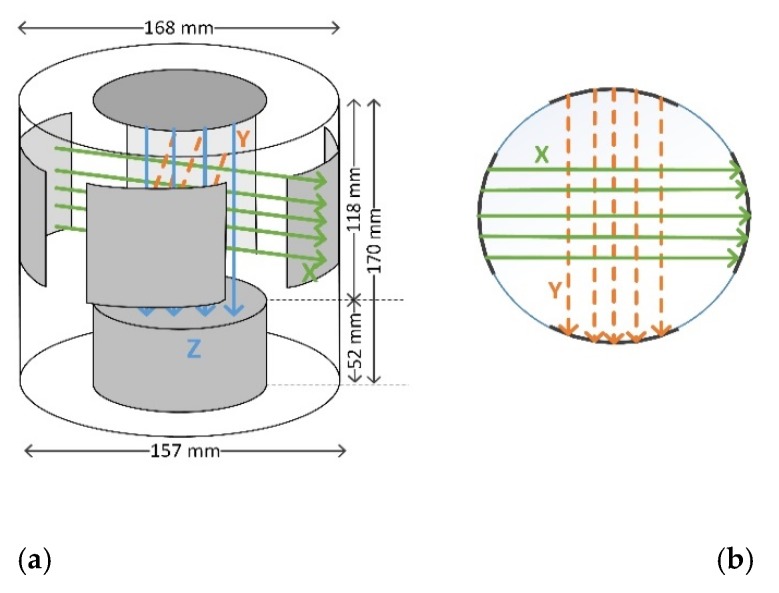
Electrode configuration: (**a**) 3D schematic, (**b**) top view.

Hioki 3531Z HiTester impedance analyser was used to measure the complex impedance spectrum from 42 Hz to 5 MHz. The total number of different frequencies were 34. The open circuit voltage was 10 V pp sine wave excitation and 16 averaging measurements were made with slow speed. Before the measurement series, open and short circuit compensations were carried out to calibrate the impedance system. Aluminum sheet electrodes were used in this study (Figure 2 and Figure 3). They were glued inside the measurement container and connected to the impedance analyser via coaxial cables. Three electrode pairs were used to measure the wood chip samples in x-, y- and z-direction. Size of the electrodes in x- and y-direction was 50 × 80 mm (height × width) and d = 100 mm in z-direction; the distance between electrodes was 118 mm in z-direction and 160 mm in x- and y-direction. The electrodes were in contact with the plastic bag including wood chip sample during the measurement and thus there was a capacitive connection between sample and electrodes. Electrodes in x and y-directions (electric field in horizontal direction) were curved and flat in z-direction (electric field in vertical direction). The measurement was carried out in a Faraday gage to reduce electromagnetic field noise.

A series of impedance measurements was conducted, including drying and re-wetting of the samples. At first, the impedance measurements were made at original condition after the sample preparation (originally fresh wood). The next impedance measurements were made when the samples were drying at the laboratory (T = 22–24 °C, RH = 17–19%) for several days. Before measurement, each sample was mixed to reduce the moisture distribution inside the sample. When the MCs of samples were below 20%, water was added into the samples to increase the MC. After adding the water, the samples were conditioned at least 24 h before the next impedance measurements. The series measurements including drying and rewetting was repeated two times. After the series, the samples were dried at +103 °C and the accurate MC was determined using weighing. Finally, the dried samples were measured again using EIS.

### 2.1. The Extractive Content Determination

The extractive contents of the samples were determined according to the method [41]. The method includes acetone extraction and gas chromatography analysis. The amount of acetone-soluble matter in wood chips provides a measure of the content of wood extractives, often called resin. The acetone-soluble matter includes, e.g., fatty acids, resin acids, fatty alcohols, sterols, di- and tri-glycerides, steryl esters and waxes. In addition, acetone-extracts of wood chips and mechanical pulps may also contain phenolic compounds such as lignans.

### 2.2. Data Analysis

**Figure 4:**
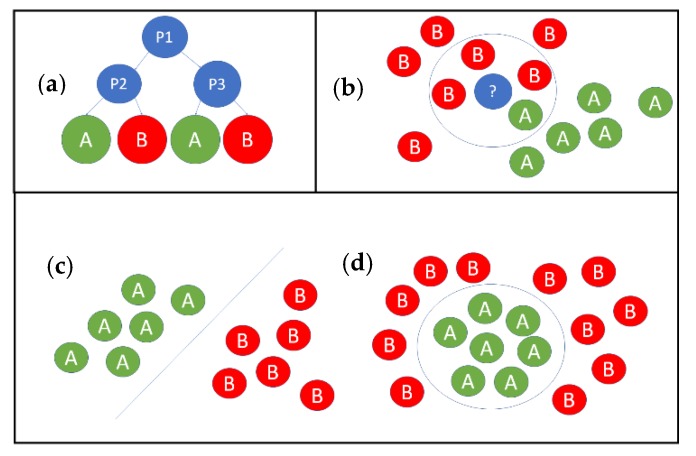
Principles of the machine learning methods: (**a**) decision tree; (**b**) KNN classifier and (**c**) linear support vector machine and (**d**) non-linear support vector machine.

Three classification methods were used: k-nearest neighbor (KNN), decision tree (DT) and support vector machines (SVM) (Figure 4). The classification methods were tested and compared by using training and testing sets. The input from the training set was fed into a classifier and the classifier was trained. After the training, the trained classifier was applied to the testing set and the correctness of the operation was determined. The tests were carried out using Matlab2016b and Classification Learner app (The MathWorks, Inc., Natick, MA, US).

K-nearest neighbor classifier is a nonparametric classifier. The training set for each class represents a class and the unknown pattern from the testing set is classified by finding the nearest neighbors from the sets of training patterns. Statistically more reliable results can be achieved by using more than one nearest neighbor. In KNN, the unknown pattern is placed in a class with most of the k-nearest neighbors in the training set.

Decision tree is a nonparametric classifier. It builds a tree-model based on the training data, where the root of the tree is the entire population of the input and each leaf represent the different classification outputs. The output leaf is selected by the decision nodes that represent different input values.

A support vector machine is a non-parametric classifier. It builds a hyperplane that maximizes the margin between the classes. Hyperplane is built based on the training observations, which are closest to different classes. Training observations, which are used to build the separating hyperplane are called support vector machines. The hyperplane can be linear or nonlinear separable.

The machine learning methods were validated using cross-validation, which separates the data to multiple training and test sets. Training sets are used to train the model and the test set is used to calculate the error of the trained model. Leave-one-out cross-validation was used, the method uses one sample as a test set and rest of the data set as a training set. Leave-one-out cross validation builds as many different models as there are samples and then calculates the average error of the models. Every sample is used once as a test set and the error is calculated based on these samples. Average accuracy of the models was used to determine the accuracy of the selected model. As leave-one-out cross-validation creates a single result for each of the models, deviation of the results was not calculated.

Neighborhood component analysis (NCA) was used to select optimal frequencies from the spectral data. Different frequencies were used for different classification sets.

Trial and error were used to choose the best hyperparameters for each of the models. Models were validated using different hyperparameters and model with the best accuracy were used as a classifier. KNN were tested using 1, 10 and 100 for number of neighbours. Euclidean distance metric function was used in all KNN models. Support vector machine models were tested using linear, polynomial and Gaussian kernel functions. One was used for a multiclass method in all support vector machine models. Decision tree models were tested using 4, 20 and 100 values for maximum number of splits. Gini index was used for split criterion method in all decision tree models. All data were standardized before training the models.

## 3. Results

Impedance spectra were measured from three different directions, all consisting of 34 different frequencies from 42 Hz to 5 MHz. The impedance modulus and phase values with standard deviations are presented at 10 kHz and 1 MHz (Table 1).

**Figure 5:**
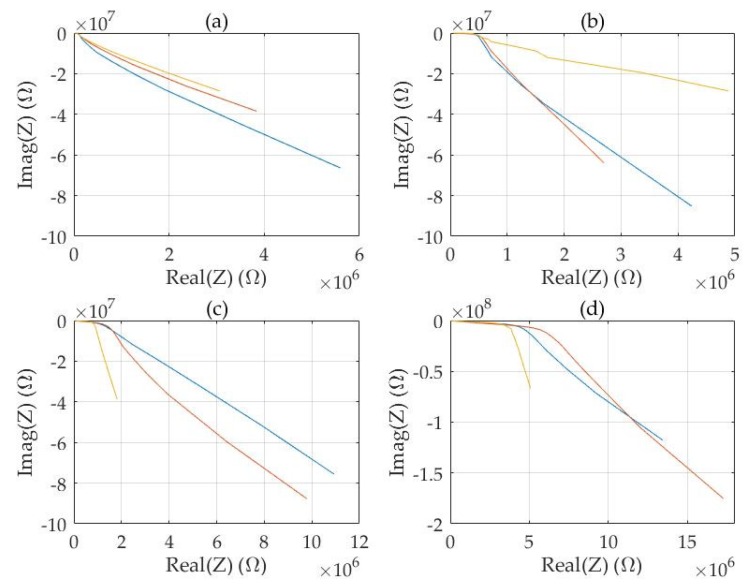
Examples of electrical complex spectra of fresh pine chips: (**a**) pine sapwood 100%, (**b**) pine sapwood 75%, heartwood 25%, (**c**) pine sapwood 25%, heartwood 75% (**d**) pine heartwood 100%. Frequency range 120 Hz–1 MHz. Measurements in x- (red), y- (blue) and z-directions (yellow, top to bottom).

**Figure 6:**
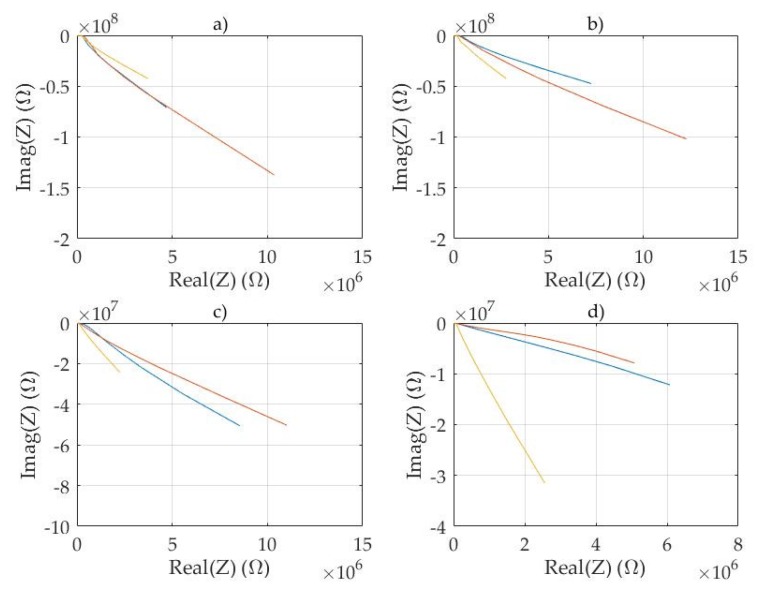
Examples of complex electrical spectra of fresh birch chips and bark: (**a**) Birch wood 100%, (**b**) birch wood 75%, bark 25%, (**c**) birch wood 25%, bark 75% and (**d**) bark 100%. Frequency range 120 Hz–1 MHz. Measurements in x- (red), y- (blue) and z-directions (yellow, top to bottom).

Examples of the measured complex electrical impedance spectra are shown for fresh pine (Figure 5) and for fresh birch (Figure 6).

The impedance spectra of pine sapwood chips and heartwood chips were considerably different (Figure 5a,d) in respect of the dispersion frequency and the magnitudes of the real and imaginary parts. For the mixed materials (Figure 5b,c) the differences in spectra were reduced but observable. The measurements at x- and y-directions (Figure 3) were quite similar but z-direction was different.

The impedance spectra of birch wood chips and birch bark were different (Figure 6a,d). Especially the ratios of the real and imaginary parts of the spectra were different. The real and imaginary part of impedance measured from bark was smaller compared to the response of stem wood chips. When spectra of mixed materials (Figure 6b,c) were measured, the difference was reduced but still the differences can be recognized from the spectra. Similar to the pine chips, the measurements at x- and y-directions were quite similar, but the z-direction was different. The difference between the complex electrical spectra measured at three different directions is remarkably different when comparing stem wood material and bark.

The three classification techniques were used to determine the efficiency of multi-parameter EIS in categorizing the raw materials. The shown accuracy is the average from each of the model results.

Several classification tests were made. Table 2 shows the classes and MC range for the tests including 75% or 100% pure material content. Classification results are shown in Table 3 and Table 4.

Classification of pure materials gave the best results, by using SVM or KNN it was possible to achieve correct classification rate better than 90% in MC range 0–60%. The best results were achieved using KNN with 10 for the number of neighbors. SVM gave the best results using polynomial kernel. When increasing the number of classes, the correct classification (%) was reduced (Table 3 and Table 4). When the MC range was above the FSP (about 30% MC), the classification results were improved, KNN classifier gave 73% classification accuracy for pine heartwood content and both KNN and SVM 66% for birch bark content. The best results were achieved using KNN with 1 for the number of neighbors. SVM gave the best results using polynomial kernel.

The results of the extractive content analyses showed that pine heartwood and birch bark contain substantial amount of extractives compared with birch wood or pine sapwood. The extractive content decreased when the material was dried, except for pine sapwood. The highest change was with birch wood samples.

## 4. Discussion

The study represents novel methods to improve analysis of biomass. So far the main interest of the studies has been moisture content using large scale of measurement methods including electromagnetic spectrum from very low frequencies [19,20] to high frequencies, e.g., gamma-rays [24]. This study showed that it is possible to classify materials according to their electrical impedance spectrum, and thus, it will be possible to determine more accurate models for MC, as one of the main issues affecting the accuracy of MC determination is the inhomogeneity of the studied material.

In the MC range, 0–60%, MC dominates the impedance measurement as expected. With narrower MC range (30–60%), which is typical MC range for industrial processes utilizing wood chips, the classification results improved significantly. It was possible to classify materials even into five classes according to heartwood/sapwood ratio or bark/stem wood ratio. In addition, the pure raw materials could be distinguished from each other using EIS with good accuracy in all MC ranges. The plastic insulator layer (plastic bag) between aluminum electrodes and the sample inhibited effectively the electrode polarization effect, which would has a high impact on the measurement. On the other hand, because of the insulation, there is no direct current going through the sample and the response is mainly capacitive. If the contact between electrodes and material would have been galvanic, accumulation of ions would have a significant effect to the results (effect of current density). By using thin plastic bags around the sample, it was possible to eliminate the electrode polarization effect, and it is more practical because plastic bags are used in industry.

The determined extractive contents were in accordance with previous studies [23]. Birch bark contains significantly more extractives than birch wood and pine heartwood, and is rich in extractives compared to pine sapwood. The effect of extractives has been reported earlier—electrical impedance spectroscopy may be used to distinguish wood materials according to their extractive content [9]. If the MC variation is high and MC is below the fiber saturation point (FSP), the MC dominates the EIS measurement. When MC is above the fiber saturation point or if the MC is limited to a certain range, the classification according to wood material’s characteristics is possible using EIS. In addition to the extractive content, other characteristics of the material affect the results, too. This is important especially for the different types of materials such as birch stem wood and bark. Cellulose, hemicellulose, lignin and ash content is also very different between stem wood and bark [23,42,43].

When comparing the heartwood/sapwood ratio at MC range 30–60% there was no pure heartwood samples at that MC range. Thus, four group classification was used. On the other hand, MC may be determined accurately using EIS, and thus, the MC value might be useful for the material analyses.

When the data was divided into smaller classes, the distributions got narrower. For analyzing materials with 2–3 classes, the distributions were workable for the classification. When the number of classes increased to five, some of the test samples differed considerably from the ones used in the training, which affected the classification results.

One of the new findings in this study was that birch material could be distinguished from pine material with good accuracy in all tested MC ranges. Birch has higher density on average, which affects the capacitance. On the other hand, the anatomical structure of hardwood and softwood is different, which affects the results. Structural differences affect the electrical properties, e.g., when measuring at different directions of wood. Here, we measured the electrical properties of samples in x-, y- and z-directions (Figure 3). The distributions of electrical parameters in different frequencies are different when measuring from top to bottom compared to the side-to-side measurements though the material is cut to chips and blended. The difference of bark from stem wood materials was detectable especially when comparing the difference of x- and y-direction with z-direction.

When comparing the classification methods, the DT classification gave the poorest results. The overall results of SVM and KNN were better and similar to each other. The inhomogeneous materials including distributions and complex non-linear data affected the results. The SVM uses vectors and KNN measurement points for classification, and both are non-linear methods. Thus, the methods produced similar classification results. If only few neighbor elements are used, KNN may handle non-linear data effectively [44]. All the studied machine learning methods could be used effectively to classify the studied material despite of the varying MC. The result shows the potential of combined electrical spectrum analysis with machine learning for advanced analysis of wood chips.

Artificial intelligence-based non-linear methods, with EIS, may be even more advantageous for industrial material analyses than the equivalent model analyses because of the efficiency of the models to extract useful non-linear information from the electrical spectra. The models may be implemented for real time measurement, which is often not possible for equivalent model analyses because of time consumption. In theory, the raw data from electrical spectra contain all information about the measurement. Even though the equivalent model analysis is perhaps the best way to make a theoretical model for an electrical system, it is not always the best choice for industrial application when quick real-time analysis is required.

EIS may be used together with the other measurement techniques. By using two or more methods together, the analysis may be improved. Machine vison and X-ray techniques are among the potential methods to increase the biomass analysis accuracy together with EIS. X-rays and EIS can measure the whole volume of biomass but machine vision systems can get information only from the surfaces. Tomographic systems, including electrical impedance tomography and/or X-ray tomography may give accurate spatial information of the biomass material.

## 5. Conclusions

Good classification efficiency was achieved by using electrical impedance spectrum analysis combined with multiple parameter analyses and machine learning techniques. It was not possible to distinguish small levels of bark or heartwood content if MC range was from 0–60% but pure materials were distinguished from each other. The results improved significantly when the MC range was limited to typical industrial process moisture contents above FSP (30–60%). An overall 73% correct classification was achieved for different degrees of pine heartwood content (four classes) when using KNN classifier. When birch bark content was studied, both KNN and SVM classifiers gave 66% correct classification rate (five classes). When pure materials were classified (two classes), KNN classifier gave 93% classification accuracy for birch bark content (MC range 0–58%), and 91% accuracy for pine heartwood content (MC range 0–58%), respectively. EIS method combined with machine learning holds potential for an advanced analysis method for wood chips for laboratory and industry.

## Figures and Tables

**Table 1:** Impedance and phase average and standard deviation values at 10 kHz and 1 MHz in pure material sets. Measurement X-direction (X), Y-direction (Y), and Z-direction (Z), (Figure 3).

Material	10 kHz		1 MHz	
	|Z| (Ω)	φ (°)	|Z| (Ω)	φ (°)
Pine sapwood (X)	6.6 (0.7)	−68.4 (13.0)	5.2 (0.3)	−63.3 (9.1)
Pine heartwood (X)	7.5 (0.5)	−67.8 (14.8)	5.8 (0.2)	−77.9 (7.9)
Birch wood (X)	6.9 (0.6)	−69.4 (10.5)	5.4 (0.3)	−69.0 (10.5)
Birch bark (X)	6.1 (0.8)	−60.4 (10.7)	4.8 (0.5)	−53.8 (11.1)
Pine sapwood (Y)	6.6 (0.7)	−67.9 (12.8)	5.2 (0.4)	−63.1 (9.2)
Pine heartwood (Y)	7.4 (0.5)	−67.3 (15.1)	5.8 (0.2)	−78.0 (7.9)
Birch wood (Y)	6.9 (0.6)	−69.4 (10.7)	5.4 (0.3)	−69.4 (9.8)
Birch bark (Y)	6.0 (0.7)	−61.1 (12.4)	4.7 (0.6)	−50.9 (13.1)
Pine sapwood (Z)	6.3 (0.7)	−63.6 (15.9)	5.1 (0.4)	−64.5 (12.1)
Pine heartwood (Z)	5.6 (0.2)	−80.5 (7.5)	5.6 (0.2)	−80.5 (7.5)
Birch wood (Z)	6.6 (0.6)	−66.7 (12.0)	5.2 (0.4)	−70.4 (11.6)
Birch bark (Z)	6.0 (0.7)	−74.5 (11.3)	4.5 (0.6)	−56.3 (13.1)

**Table 2:** Classification of heartwood content from pine chips and bark content from birch chips: material classes according to the content percentages and moisture content range of each class.

Material Test	Class 1	Class 1 MC-Range (%)	Class 2	Class 2 MC-Range (%)
Pine100	heartwood	0–24	sapwood	0–58
Pine75	heartwood 75–100%	0–31	Sapwood 75–100%	0–58
Birch100	birch bark	0–58	birch wood	0–46
Birch 75	birch bark 75–100%	0–58	birch wood 75–100%	0–48

**Table 3:** Classification results, correct classification (%). Classification of heartwood content from pine chips, bark content from birch chips and pine chips from birch chips and birch bark. MC range 0–60%. Test groups and correct classification using decision tree (DT), support vector machine (SVM) and K-nearest neighbor (KNN). N = number of samples. Birch/pine classification included three classes: birch, pine and birch bark.

Material Test	DT (%)	SVM (%)	KNN (%)	N
Pine100	73	85	90	41
Pine75	69	85	89	80
Birch100	89	93	93	53
Birch 75	74	83	75	101
Birch/pine100	65	78	69	80
Birch/pine 75	71	84	86	152

**Table 4:** Classification results, correct classification (%). Pine heartwood/sapwood and birch bark/wood, 5 classes: 0, 25%, 50%, 75% and 100% mix ratios. Classification by using decision tree (DT), support vector machine (SVM) and K-nearest neighbor (KNN). N = number of samples.

Material/ MC Range (%)	DT (%)	SVM (%)	KNN (%)	N
Pine				
0-60	41	35	30	97
30-60 *	30	65	73	40
Birch				
0-60	34	40	37	123
30-60	61	66	66	61

* Four classes—MC of heartwood samples was below 30%.
